# An unresectable and metastatic intrahepatic cholangiocarcinoma with EML4-ALK rearrangement achieving partial response after first-line treatment with ensartinib: a case report

**DOI:** 10.3389/fonc.2023.1191646

**Published:** 2023-08-22

**Authors:** Senmiao Huang, Dianhe Li, Yongye Huang, Guojie Lu, Ying Tian, Xuefeng Zhong, Yating Zheng, Mengli Huang, Fuxi Huang

**Affiliations:** ^1^ Oncology Department, Guangzhou Panyu Central Hospital, Guangzhou, China; ^2^ Digestive Center Area Two, Guangzhou Panyu Central Hospital, Guangzhou, China; ^3^ Thoracic Surgery, Guangzhou Panyu Central Hospital, Guangzhou, China; ^4^ Pathology Department, Guangzhou Panyu Central Hospital, Guangzhou, China; ^5^ The Medical Department, 3D Medicines Inc., Shanghai, China

**Keywords:** intrahepatic cholangiocarcinoma (ICC), EML4-ALK rearrangement, ALK fusion, ensartinib, targeted therapy

## Abstract

Systemic chemotherapies are the primary treatment options for patients with unresectable and metastatic intrahepatic cholangiocarcinoma (ICC), but the effectiveness of current systemic therapies is limited. The development of targeted-therapy has changed the treatment landscape of ICC, and comprehensive genome sequencing of advanced cholangiocarcinoma patients could be beneficial to identify potential targets to guide individualized treatment. Herein, we reported an unresectable and metastatic ICC patient who detected EML4-ALK rearrangement in peripheral blood, which was later confirmed on tissue-based testing, and achieved partial response (PR) after first-line treatment with ensartinib. This case suggests that the liquid biopsy is of clinical value for unresectable or metastatic ICC, and the discovery of rare molecular targets provides new therapeutically approaches for advanced ICC patients.

## Introduction

Cholangiocarcinoma is a malignant tumor with features of cholangiocyte differentiation. According to anatomical location, cholangiocarcinoma is divided into intrahepatic, perihilar, or distal cholangiocarcinoma (1). Intrahepatic cholangiocarcinoma (ICC) accounts for less than 10% of cholangiocarcinoma cases, compared with 50% in perihilar cholangiocarcinoma and 40% in distal cholangiocarcinoma (2). However, the incidence of ICC has increased globally in recent years, and the incidence in Asian population is significantly higher than that in European and American population (3). Surgery is the preferred option for all cholangiocarcinoma patients, but approximately 65% of patients are difficult to perform surgical treatment because of local invasion or distant metastasis at initial diagnosis (1, 2). According to relevant data, 26.4% of ICC patients have distant metastasis at initial diagnosis (4). The most common metastasis sites are the multiple metastasis (9.1%), the liver only (5.2%), the distant lymph node only (3.8%), and the lung only (2.4%), respectively. And the multi-metastasis sites are mainly liver plus lung (7.1%), and liver plus distant lymph node (6.2%) (4). The prognosis of different metastatic sites is also different, among which multi-metastasis displays the shortest survival (4).

For patients with unresectable and metastatic ICC, systemic chemotherapies are the primary treatment options (1, 2). However, the effectiveness of current systemic therapies is limited: the median overall survival (OS) of gemcitabine combined with cisplatin in first-line therapy was only 11.8 months (1). In recent years, the development of targeted-therapy has changed the treatment landscape of malignancies, but the progress in cholangiocarcinoma has been modest (1, 2). Comprehensive genome sequencing of advanced cholangiocarcinoma patients could be beneficial to identify potential targets to guide individualized treatment. Herein, we report an advanced ICC patient with multiple metastases (including liver, lung, and mediastinal lymph nodes) who detected EML4-ALK rearrangement and achieved partial response (PR) after first-line treatment with ensartinib, and the progression-free survival (PFS) had lasted for 6.3 months at the latest follow-up time.

## Case presentation

**Figure 1:**
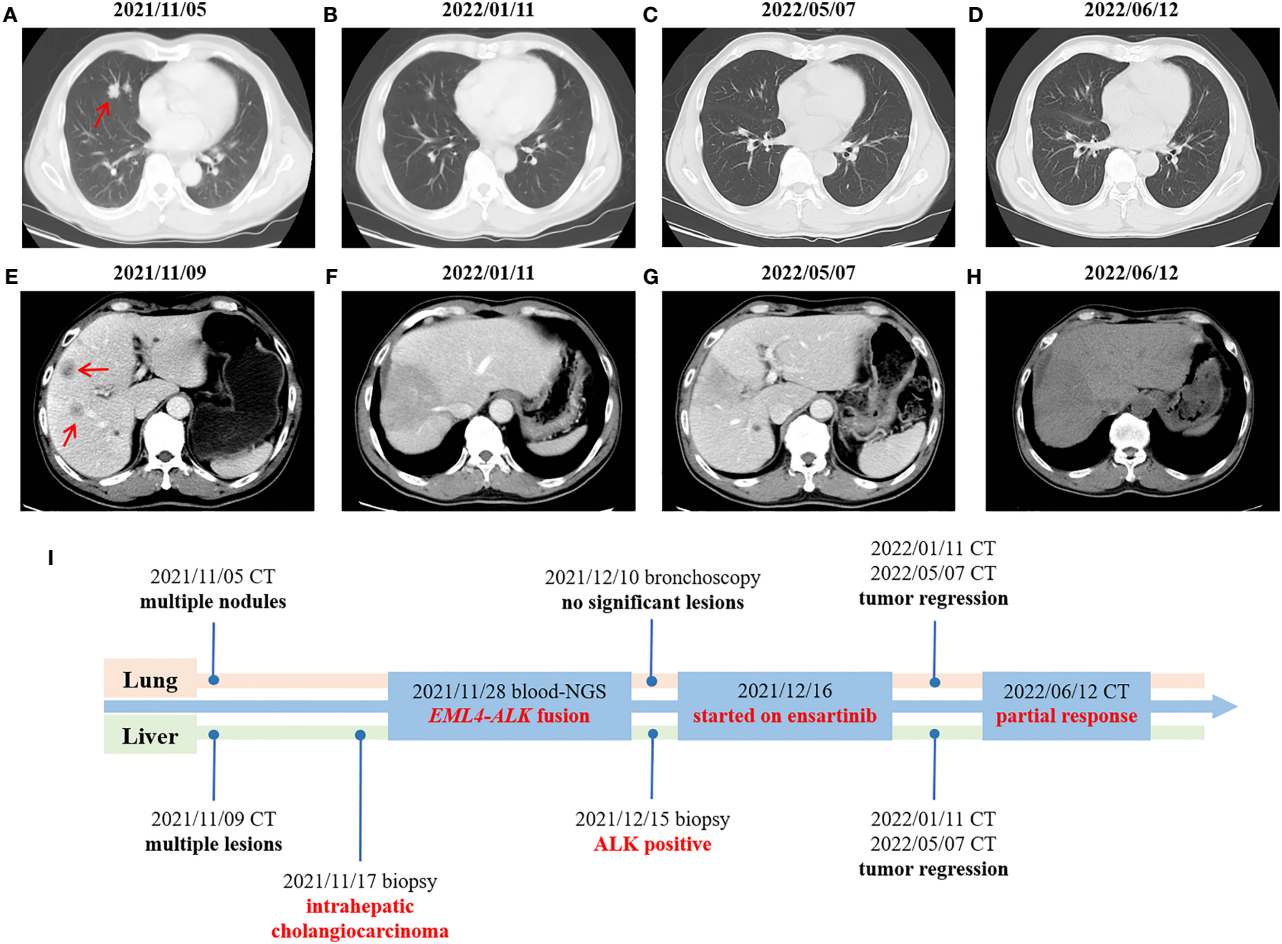
Changes in imaging throughout the treatment period. **(A–D)** CT scans of the chest at different times. **(E–H)** CT scans of the abdomen at different times. **(I)** Timeline of treatment process. Red arrow: the location of the tumor lesion.

**Figure 2:**
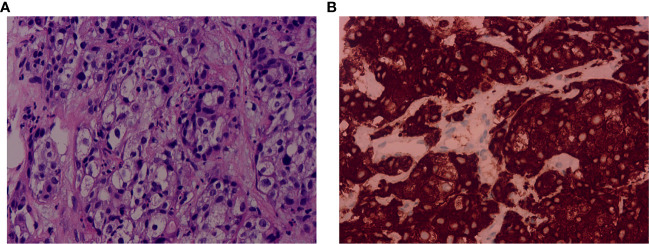
Pathological examination of the liver biopsy and immunohistochemical staining of ALK expression. **(A)** The pathological examination showed poorly differentiated adenocarcinoma. Hematoxylin and eosin (H&E) stain. Magnification: 40×. **(B)** The immunohistochemical result showed positive for ALK (D5F3). Magnification: 40×. ALK, anaplastic lymphoma kinase.

A 67-year-old male was admitted to hospital on November 5th, 2021 due to persistent fever, chest tightness and persistent pressure behind the sternum without obvious inducement. He had no history of smoking or drinking, and denied any history of hepatitis or family history. On November 5th, 2021, a chest computed tomography (CT) scan showed two nodules in the medial segment of the middle lobe of the right lung (the larger one was about 15×20mm), and there were enlarged lymph nodes in the right hilar and mediastinum (the larger one was 30×33mm), which was considered to be lung cancer around the middle lobe of the right lung and lymph node metastasis in the right hilar and mediastinum (**Figure 1A**). The abdominal enhanced CT in November 9th, 2021 showed multiple low-density space-occupying lesions of different sizes in the liver, with the largest lesion located in the S8 segment of the liver, about 105×112mm in size (**Figure 1E**). In addition, there were other low-density occupying sites in the liver, and the diameter of the larger lesion was about 20mm. (**Figure 1E**). The main clinical diagnosis and treatment process of the patient is shown in **Figure 1I**. Ultrasound-guided needle biopsy of liver mass was performed in November 17th, 2021, the pathological results were considered to be poorly differentiated adenocarcinoma (**Figure 2A**). Immunohistochemistry (IHC) indicated the sample being positive for CK, CK7, P40 (partially positive), TTF1 (weak positive), CK19, Ki-67 (about 20%+), CD10, MUC-1, GATA-3, and negative for CK20, NapsinA, hepatocyte marker, Arginase-1, Glypican3, CD34, ER (**Supplementary Figure 1**). The light microscopic morphology and immunohistochemical results tended to be ICC. In addition, IHC for PD-L1 was negative (TPS<1%, CPS<1) (**Supplementary Figure 1**). On November 26, 2021, painless colonoscopy and painless gastroscopy were performed. The mucous membranes of cecum, ascending colon, transverse colon, descending colon, sigmoid colon and rectum were smooth, the blood vessels were clear, and no abnormal secretions, new organisms or erosions were found (**Supplementary Figure 2A**). Gastroscopy showed no obvious lesions (**Supplementary Figure 2B**).

Because the lung lesions are too small to be biopsied, the diagnostic basis of lung cancer is insufficient. On November 28th, 2021, the patients’ peripheral blood sample was collected and used for the extraction of circulating free DNA (cfDNA). A 733-gene panel was used for next generation sequencing (NGS) analysis of cfDNA. Upon NGS analysis result, the patient was identified to harbor EML4-ALK rearrangement (**Supplementary Figure 3**), and *STK11* mutation (exon5 p.T209Gfs*60). Another liver biopsy was performed on December 15th, 2021, and IHC results showed positive for ALK (D5F3) (**Figure 2B**). The results of hematological examination on December 16, 2021 showed that C-reaction protein (132.43 mg/L), leukocytes (52.79×10^9/L), neutrophils (49.45×10^9/L), monocytes (2.57×10^9/L), platelets (392×10^9/L), glucose (7.03 mmol/L) and procalcitonin (18.63 ng/ml) levels increased significantly, while the levels of lymphocytes (0.74×10^9/L), erythrocytes (3.28×10^12/L), hemoglobin (64 g/L), mean RBC hemoglobin (19.4 pg), sodium (127.1 mmol/L) and chlorine (93.1 mmol/L) decreased significantly. In addition, liver function index examination showed the levels of aspartate aminotransferase (73 U/L) and glutamyl transpeptidase (323 U/L) were increased (**Supplementary Table 1**). Combined with the above findings, the patient was diagnosed with ICC. Chemotherapy was not given because of the patient’s strong rejection. The patient was subsequently started on ensartinib (225mg P.O qd) at first-line therapy on December 16th, 2021, considering that the NGS test showed an EML4-ALK rearrangement and the IHC showed a positive ALK. A follow-up CT scan conducted approximately 1 months after treatment onset on January 11, 2022 demonstrated obvious tumor regression, compared with the results on November 5th, 2021, with a decrease in nodule in the medial segment of the middle lobe of the right lung from 15×20 mm to 12×15 mm, and lymph node in the right hilar and mediastinum from 30×33mm to 20×15 mm (**Figure 1B**). Compared with November 9, 2021, the lesion at the S8 segment of the liver was reduced from 105×112mm to 69×96mm, and other low-density space occupying lesions in the liver were reduced from 20mm to 12mm (**Figure 1F**). Hematological examination results on January 10, 2022 showed C-reactive protein (13.68 mg/L), leukocytes (9.27×10^9/L), neutrophils (56.1×10^9/L), monocytes (0.76×10^9/L), platelets (391×10^9/L, glucose (4.82 mmol/L) and procalcitonin (0.2 ng/ml) were significantly decreased, and the levels of leukocytes, neutrophils and glucose were reduced to the normal range, while the levels of lymphocytes (1.81×10^9/L), erythrocytes (4.51×10^12/L), and chlorine (103.5 mmol/L) rose to normal levels. The levels of aspartate aminotransferase (41 U/L) and glutamyl transpeptidase (80 U/L) were also decreased (**Supplementary Table 1**). Based on the above results, the patient was considered to have PR. The patient reported no adverse events during the treatment. Therefore, the patient continued to receive ensartinib, and a CT scan on May 7, 2022 revealed continued focal shrinkage and PR status compared to January 11, 2022. Specifically, the nodule in the medial segment of the middle lobe of the right lung was reduced from 12 × 15 mm to 5 × 3 mm, lymph node in the right hilar and mediastinum was reduced from 20×15 mm to 13×12 mm (**Figure 1C**), and the lesion at the S8 segment of the liver was reduced from 69×96mm to 58×75mm (**Figure 1G**). These indicated that the patient had a good response to ensartinib treatment. Compared with before treatment, the levels of the above hematological indicators are closer to the normal level (**Supplementary Table 1**). Therefore, the patient continued to receive ensartinib, and follow-up CT examination was carried out on June 12, 2022, the results showed that the patient maintained PR status (**Figures 1D, H**). The main clinical diagnosis and treatment process of the patient is shown in **Figure 1I**.

## Discussion

Surgical resection is the only curative treatment for ICC, but quite a few patients have no chance for surgery due to distant metastasis at initial diagnosis (4), so that proper systemic treatments are crucial for advanced ICC patients. However, there are few systemic treatment options available for advanced ICC patients. Gemcitabine-based or fluoropyrimidine-based chemotherapy remains the dominant systemic treatment of cholangiocarcinoma, but the effectiveness of chemotherapy for advanced ICC is limited (1). Molecular profiling studies have delineated the genomic landscape of cholangiocarcinoma and revealed about 40% of patients harbor potentially targetable genetic driver alterations (5). Even so, there are few molecularly targeted drugs approved for ICC. In this case, the EML4-ALK rearrangement was detected in peripheral blood of an advanced ICC patient with multiple metastases, which was later confirmed on tissue-based testing. This patient was treated with ensartinib at the first line, and achieved PR after 3 months of treatment.

Anaplastic lymphoma kinase (ALK) was first named for its role as a fusion partner for chromosomal translocation in anaplastic large cell lymphoma (6). A variety of ALK gene alterations have been described across many tumor types, including single nucleotide variants, deletions and rearrangements (6). ALK rearrangements, also named ALK translocations or fusions, are an established targetable alteration in around 5% of non-small cell lung cancers (NSCLC) patients, of which the EML4-ALK rearrangement is the most common (6). The resultant ALK fusion protein is a validated target in NSCLC, and lots of ALK tyrosine kinase inhibitors (TKIs) are currently approved in ALK-positive NSCLC (7). In a network meta-analysis study, the treatment efficacy of ALK-TKIs (such as alectinib, brigatinib, ensartinib, and lorlatinib) in Asian ALK-positive NSCLC patients was compared and the result showed that ensartinib might be the most effective first-line treatment for Asian ALK-positive NSCLC patients (8).

Currently, the clinical evidence of ALK fusion proteins in cancer largely originates from ALK-positive NSCLC, but there are scarce data regarding the incidence and targeted therapy of ALK rearrangements in cholangiocarcinoma. Several studies showed that ALK rearrangements are found in <1% of cholangiocarcinoma patients (9–11). A retrospective analysis evaluated the predictive significance of ALK, c-ros oncogene1 receptor tyrosine kinase (ROS1), or mesenchymal to epithelial transition (MET) aberrant expression (RAM) in advanced or metastatic biliary tract cancer patients treated with gemcitabine plus oxaliplatin with or without cetuximab, and found that all RAM-high (immunohistochemistry intensity 3+ for any markers) tumors derived from ICC patients, who had shorter OS than RAM-low (immunohistochemistry intensity <3+ for all markers) ICC patients (median OS, 5.7 vs. 11.7 months, P=0.021) (12). Sporadic cases of cholangiocarcinoma with ALK rearrangement have been recently reported (13–15), among which two patients were confirmed to harbor EML4-ALK rearrangement, but not treated with ALK-TKIs (13, 14). Another case described an ICC patient with STRN-ALK rearrangement, who responded to either alectinib (a response lasting for 7 months) or lorlatinib (a response lasting for 3 months) after the failure of standard chemotherapy at the first and second lines treatment (15). In addition, there were four phase II clinical trials (NCT02374489, NCT02638909, NCT02034981, and NCT02568267) assessing the efficacy of different TKIs (crizotinib, ceritinib, or entrectinib) in advanced solid tumors, including cholangiocarcinomas, all of which harbored ALK, ROS1, or neurotrophin receptor kinase (NTRK) rearrangements. A phase I study evaluated the maximum tolerated dose of ceritinib combined with gemcitabine-based chemotherapy in advanced solid tumors patients, and showed that three out of five cholangiocarcinoma patients with ALK rearrangement had the clinical benefit (16).

In this case, multiple nodules in both lungs and intrahepatic lesions were observed at initial diagnosis, but the nodule in each lung was too small to be punctured, so that only intrahepatic lesion tissue samples could be obtained and confirmed as ICC through pathological examination. Obviously, it is difficult to determine whether the lung lesion is primary or metastatic. Therefore, the EML4-ALK rearrangement was initially detected in ctDNA from plasma, which may originate from ICC or pulmonary lesions (lung cancer) or both. Fortunately, the expression of ALK fusion protein of the intrahepatic lesion tissue was positive confirmed by immunohistochemistry, indicating that part or all of EML4-ALK rearrangements detected in ctDNA originated from ICC. However, we cannot rule out the possibility of primary lung cancer based on the available clinical information, particularly ALK rearrangement is a common driver factor in NSCLC. The incidence of synchronous double primary ICC and another cancer is too low to have sufficient data. Several cases of double primary hepatic cancers with hepatocellular carcinoma and ICC were reported (17–19), but only one case report described double primary cancers with ICC and lung squamous cell carcinoma (20). Considering the rarity of double primary cancers, we suspected that pulmonary lesions were more likely to be metastatic, but we could not obtain the lung tissue, which is a limitation of this case report.

To sum up, this case report described an advanced ICC patient with multiple metastases who harbored EML4-ALK rearrangement and achieved a PR after first-line treatment with ensartinib. The EML4-ALK rearrangement was initially detected in plasma ctDNA, suggesting that the liquid biopsy is of clinical value for advanced ICC, especially for patients with multiple metastases. Besides, attention should be paid to the efficacy of ALK-TKIs in ALK-positive ICC patients in the future.

## Data availability statement

The raw data supporting the conclusions of this article will be made available by the authors, without undue reservation.

## Ethics statement

The studies involving humans were approved by ethics committee of Panyu Central Hospital. The studies were conducted in accordance with the local legislation and institutional requirements. The participants provided their written informed consent to participate in this study. Written informed consent was obtained from the individual(s) for the publication of any potentially identifiable images or data included in this article.

## Author contributions

SH and DL were involved in the collation and interpretation of clinical data. YH and GL were involved in the interpretation of images and the discussion of treatment. YT was involved in the interpretation of pathological results. XZ and YZ were involved in the designing and drafting of the manuscript. MH was involved in the manuscript revision. FH was involved in the management of the patient. All authors contributed to the article and approved the submitted version.

## Supplementary material

Supplementary Figure 1 Other immunohistochemical results. Positive for CK, CK7, P40 (partially positive), TTF1 (weak positive), CK19, Ki-67 (about 20%+), CD10, MUC-1, GATA-3, and negative for CK20, NapsinA, hepatocyte marker, Arginase-1, Glypican3, CD34, ER, PD-L1 (TPS<1%, CPS<1).

Supplementary Figure 2 Gastroenteroscopy results. **(A)** The results of the colonoscopy. **(B)** The gastroscopy results.

Supplementary Figure 3 NGS analysis of peripheral blood sample showing *EML4-ALK* rearrangement. NGS, next generation sequencing.
