# Supplementary figures and images for: An unresectable and metastatic intrahepatic cholangiocarcinoma with EML4-ALK rearrangement achieving partial response after first-line treatment with ensartinib: a case report
Source: Front Oncol. 2023 Aug 22;13:1191646. doi: 10.3389/fonc.2023.1191646 (PMC10477974; doi:10.3389/fonc.2023.1191646)

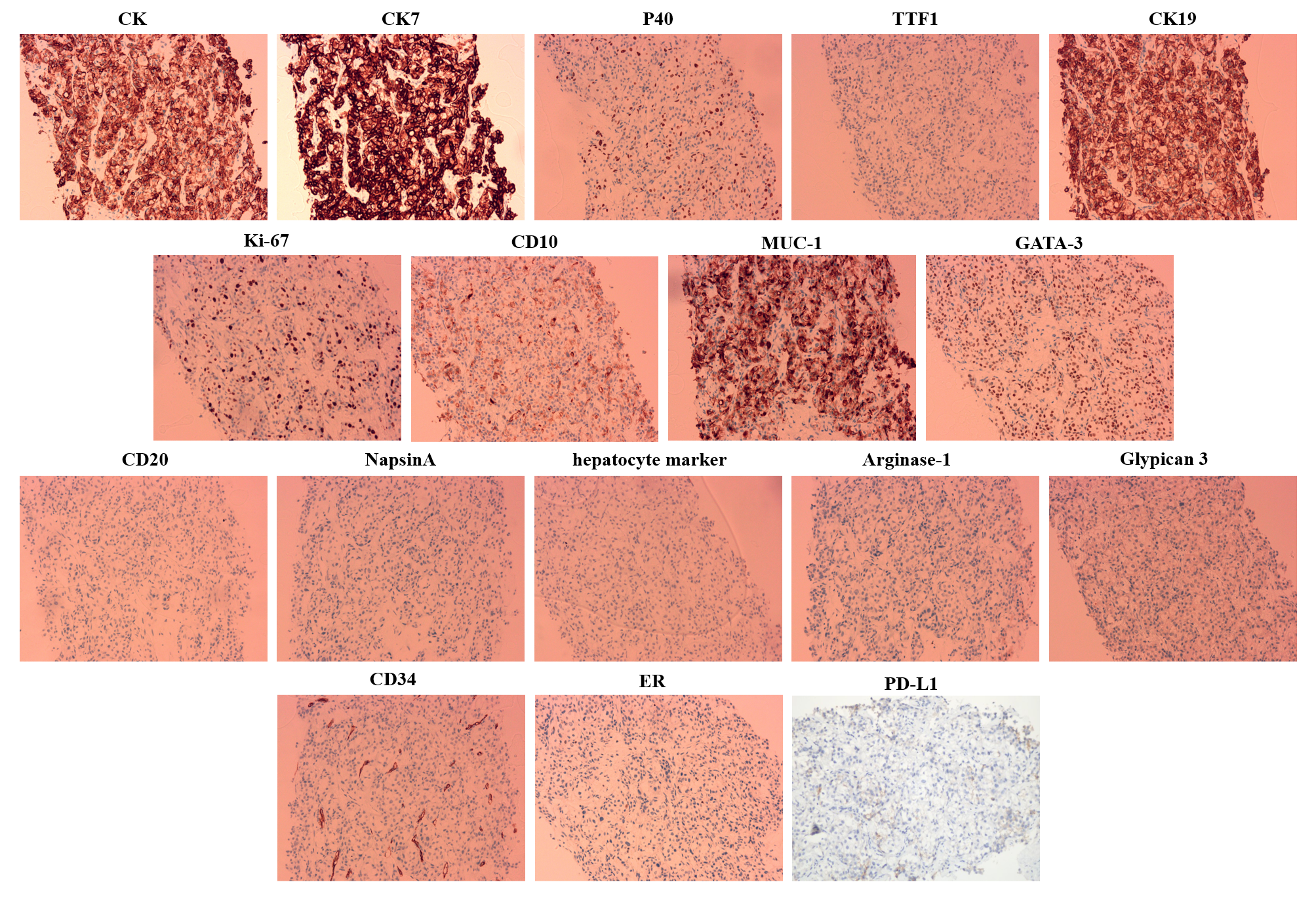

Supplement: Supplementary Figure 1 — Other immunohistochemical results. Positive for CK, CK7, P40 (partially positive), TTF1 (weak positive), CK19, Ki-67 (about 20%+), CD10, MUC-1, GATA-3, and negative for CK20, NapsinA, hepatocyte marker, Arginase-1, Glypican3, CD34, ER, PD-L1 (TPS<1%, CPS<1). [file Image_1.jpeg]

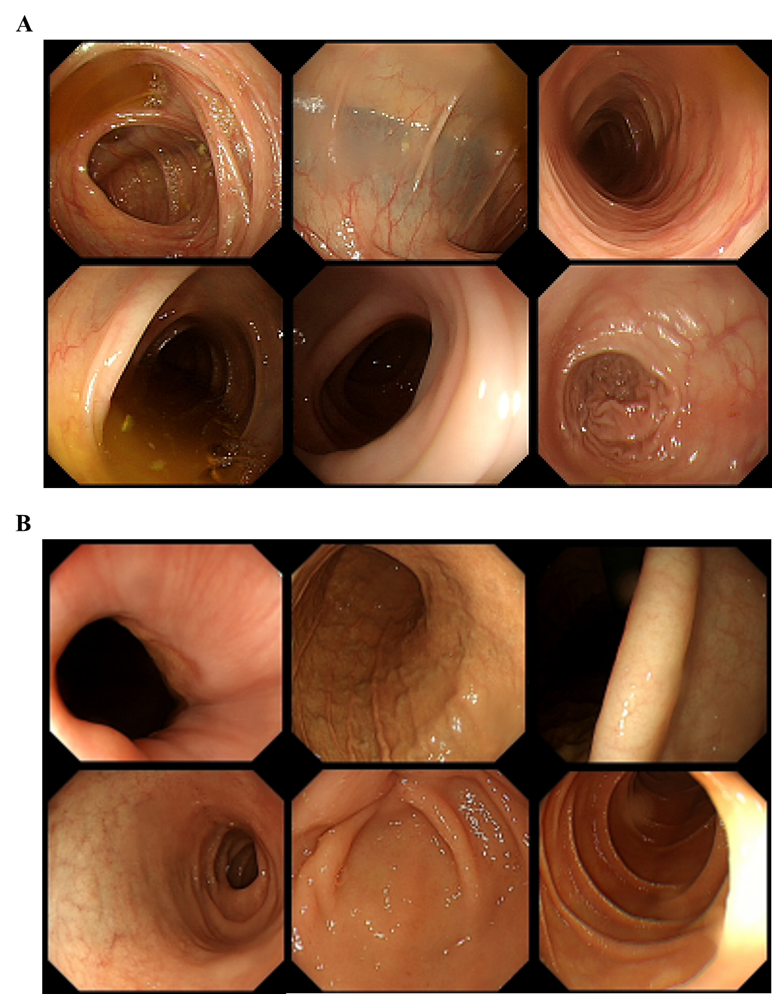

Supplement: Supplementary Figure 2 — Gastroenteroscopy results. (A) The results of the colonoscopy. (B) The gastroscopy results. [file Image_2.jpeg]

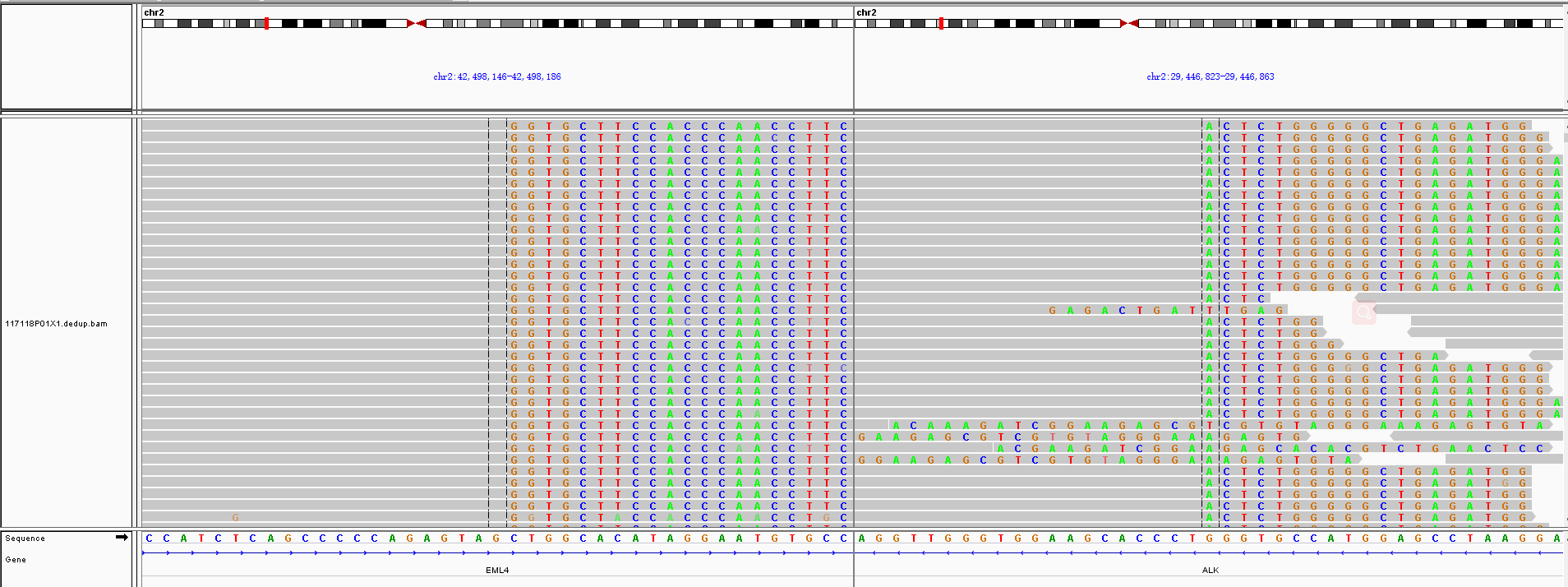

Supplement: Supplementary Figure 3 — NGS analysis of peripheral blood sample showing EML4-ALK rearrangement. NGS, next generation sequencing. [file Image_3.jpeg]
