# Supplementary material for: An unresectable and metastatic intrahepatic cholangiocarcinoma with EML4-ALK rearrangement achieving partial response after first-line treatment with ensartinib: a case report
Source: Front Oncol. 2023 Aug 22;13:1191646. doi: 10.3389/fonc.2023.1191646 (PMC10477974; doi:10.3389/fonc.2023.1191646)
Supplement: Supplementary file 4 [file Table_1.pdf]

Test items (unit)	Reference interval	Test time and results				
Blood routine index & rapid C-reaction protein		2021/12/16	2022/1/10	2022/5/10	2022/5/14	2022/6/12
C-reaction protein (mg/L)	0-10	132.43	13.68	84.6	30.36	76.59
leukocyte (10^9/L)	3.5-9.5	52.79	9.27	17.57	10.29	16.34
neutrophil (%)	40-75	93.7	56.1	83.2	78.9	81.5
lymphocyte (%)	20-50	1.4	19.5	7	11	7.4
monocyte (%)	3~10	4.9	8.2	8.9	6.9	9.8
eosinophil (%)	0.4-0.8	0	15.4	0.7	2.9	1.2
basophil (%)	0-1	0	0.8	0.2	0.3	0.1
neutrophil (10^9/L)	1.8-6.3	49.45	5.2	14.62	8.12	13.31
lymphocyte (10^9/L)	1.1-3.2	0.74	1.81	1.23	1.13	1.21
monocyte (10^9/L)	0.1-0.6	2.57	0.76	1.57	0.71	1.6
eosinophil (10^9/L)	0.02-0.52	0.02	1.43	0.12	0.3	0.2
basophil (10^9/L)	0-0.06	0.01	0.07	0.03	0.03	0.02
erythrocyte (10^12/L)	4.3-5.8	3.28	4.51	3.99	3.85	3.79
hemoglobin (g/L)	130-175	64	89	82	80	81
erythrocyte (%)	40-50	19.2	29.5	24.8	24.6	24.8
Mean erythrocyte (fl)	82-100	58.6	65.4	62.3	63.9	65.3
Mean RBC hemoglobin (pg)	27-34	19.4	19.6	20.5	20.9	21.5
Mean RBC hemoglobin (g/L)	316-354	331	300	329	327	329
Erythrocyte volume distribution width SD (%)	39-46	34.3	51.5	34.3	34.4	37.8
Erythrocyte volume distribution width CV (%)	11.7-14.4	16.4	22.3	15.3	15.1	15.7
platelet (10^9/L)	125-350	392	391	371	464	284
Mean platelet (fl)	5.1-11.1	8.4	8.8	8.5	8.4	9.2
Mean platelet (%)	0.05-0.36	0.329	0.345	0.316	0.39	0.261
platelet distribution width (fl)		14.9	14.8	15.2	15	15.6
Large platelet ratio (%)	13-43	15.5	18.7	17.3	15.4	21.6
Emergency biochemistry		2021/12/16	2022/1/10	2022/5/10	2022/5/14	2022/6/12
Potassium (mmol/L)	3.5-5.3	3.84	4.45	4.02	3.86	3.9
Sodium (mmol/L)	137-147	127.1	136.7	129.4	139.3	129
Chlorine (mmol/L)	99-110	93.1	103.5	97.1	103.6	99
Total calcium (mmol/L)	2.00-2.60	2.13	2.34	2.19	2.29	/
Carbamide (mmol/L)	2.8-8.2	4.4	5.8	4.3	4.4	5
Creatinine (umol/L)	44-133	116	130	132	150	167
Glucose (mmol/L)	3.90-6.10	7.03	4.82	7	3.72	/
Carbon dioxide (mmol/L)	22-32	24.4	26.1	23.5	25.3	/
PCT		2021/12/16	2022/1/10	2022/5/10	2022/5/14	2022/6/12
Procalcitonin (ng/ml)	0-0.05	18.63	0.2	0.42	0.19	0.56
Liver function & protein		2021/12/16	2022/1/10	2022/5/10	2022/5/14	2022/6/12
Alanine transaminase (U/L)	9-50	27	42	26	22	38
Aspartate aminotransferase (U/L)	15-40	73	41	30	22	37

Glutamate/Glutamate Transaminase ratio		0.37	1.02	0.87	1	1.03
Glutamyl transpeptidase (U/L)	10-60	323	80	97	85	133
Total protein (g/L)	65-85	56.9	74.6	/	62.1	/
Albumin (g/L)	40-55	27.3	35.5	/	29.5	/
Globulin (g/L)	20-40	29.6	39.1	/	32.6	/
Albumin/globulin ratio	1.2-2.4	0.9	0.9	/	0.9	/
Prealbumin (g/L)	0.2-0.4	0.05	0.24	/	0.15	/
Transferrin (g/L)	1.70-3.40	0.93	2.2	/	1.7	/
